# Vision Transformers in Image Restoration: A Survey

**DOI:** 10.3390/s23052385

**Published:** 2023-02-21

**Authors:** Anas M. Ali, Bilel Benjdira, Anis Koubaa, Walid El-Shafai, Zahid Khan, Wadii Boulila

**Affiliations:** 1 Robotics and Internet-of-Things Laboratory, Prince Sultan University, Riyadh 12435, Saudi Arabia; 2 Department of Electronics and Electrical Communications Engineering, Faculty of Electronic Engineering, Menoufia University, Menouf 32952, Egypt; 3 SE & ICT Laboratory, LR18ES44, ENICarthage, University of Carthage, Tunis 1054, Tunisia; 4 Security Engineering Laboratory, Computer Science Department, Prince Sultan University, Riyadh 11586, Saudi Arabia; 5 RIADI Laboratory, University of Manouba, Manouba 2010, Tunisia

**Keywords:** vision transformer, transformer, self-attention, image restoration, image super-resolution, image denoising, general image enhancement, JPEG compression artifact reduction, image deblurring, removing adverse weather conditions, image dehazing

## Abstract

The Vision Transformer (ViT) architecture has been remarkably successful in image restoration. For a while, Convolutional Neural Networks (CNN) predominated in most computer vision tasks. Now, both CNN and ViT are efficient approaches that demonstrate powerful capabilities to restore a better version of an image given in a low-quality format. In this study, the efficiency of ViT in image restoration is studied extensively. The ViT architectures are classified for every task of image restoration. Seven image restoration tasks are considered: Image Super-Resolution, Image Denoising, General Image Enhancement, JPEG Compression Artifact Reduction, Image Deblurring, Removing Adverse Weather Conditions, and Image Dehazing. The outcomes, the advantages, the limitations, and the possible areas for future research are detailed. Overall, it is noted that incorporating ViT in the new architectures for image restoration is becoming a rule. This is due to some advantages compared to CNN, such as better efficiency, especially when more data are fed to the network, robustness in feature extraction, and a better feature learning approach that sees better the variances and characteristics of the input. Nevertheless, some drawbacks exist, such as the need for more data to show the benefits of ViT over CNN, the increased computational cost due to the complexity of the self-attention block, a more challenging training process, and the lack of interpretability. These drawbacks represent the future research direction that should be targeted to increase the efficiency of ViT in the image restoration domain.

## 1. Introduction

Image Restoration is an umbrella term incorporating many computer vision tasks. These approaches seek to generate a better version of an image that is retrieved in a lower-quality format. The leading seven tasks of image restoration are: Super-Resolution, Image Denoising, General Image Enhancement, JPEG Compression Artifact Reduction, Image Deblurring, Removing Adverse Weather Conditions, and Image Dehazing. All these tasks share among them many common characteristics. First of all, they are image generation tasks. This means that we generate new data that tries to mimic the authentic representation of the information being captured inside the image. Second, they all try to remove a specific type of corruption applied to the image. This corruption may be a noise, a bad quality image sensor, a capturing imperfection, a weather condition that hides some information, etc. Third, all of them are now becoming based on deep learning techniques. This architectural change is due primarily to their effectiveness compared to old techniques. Hence, any advancement in deep learning theory directly affects these tasks and brings new ideas for any image restoration researcher.

### 1.1. Techniques Used in Image Restoration

Since deep learning popularity began in the computer vision field in 2012 [1], Convolutional Neural Networks (CNN) have been the default feature extractor used to learn patterns in the data. An overwhelming number of models and architectures have been designed and improved over the years to increase the learning capability of CNNs. Generic architectures, as well as task-specific architectures, were designed. Before 2020, it was the rule for any image restoration researcher to customize a model based on CNN to increase the efficiency of the state-of-the-art works on his task [2,3,4,5]. This approach is adopted in most computer vision tasks [6,7,8,9,10,11,12,13,14].

Since 2017, the Transformer architecture has made a significant breakthrough in the Natural Language Processing (NLP) domain. The self-attention mechanism and the new proposed architecture have proven to have a considerable ability to manage sequential data efficiently. Nevertheless, adoption in the computer vision domain was delayed until 2020, when the Vision Transformer (ViT) architecture was first introduced [15]. Up to now, ViT has proven efficient and competed very well with CNN. Here also, an overwhelming number of architectures inspired by ViT have been introduced with an attractive efficiency. Moreover, ViT has been demonstrated to solve many issues traditionally associated with CNN. Therefore, a debate between these two architectures’ advantages and drawbacks has emerged to decide where to go in future research directions.

**Figure 1:**
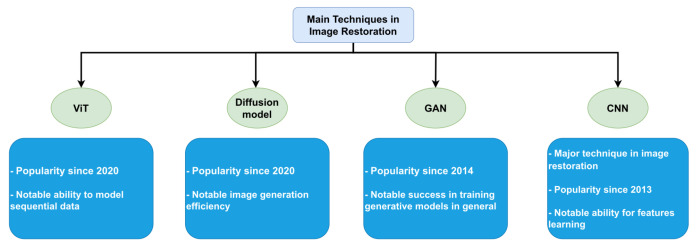
The main techniques used in Image Restoration.

Many techniques have been developed for image restoration, including those based on CNNs and ViTs. However, CNNs have several limitations regarding their ability to learn a mapping from degraded images to reach their original counterparts. In addition, the quality of the images generated by CNNs is often far from the original images. In contrast, some techniques address these challenges of CNN. These techniques include the Generative Adversarial Networks (GANS) [16] and the Diffusion model [17], as shown in Figure 1. GANs are a class of deep learning models that involve training two neural networks, a generator, and a discriminator, to work together to generate new, synthetic data similar to the real data. Despite the success of GANs in various tasks, their application in image restoration methods has often been found to produce suboptimal results due to issues such as pattern collapse, excessive smoothing, artifacts, and training instability. The Diffusion model, on the other hand, is a generative model that uses a diffusion process to generate new images by modeling the image formation process. However, while diffusion models have been used in image generation and denoising tasks, they may struggle to generate images with accurate global features and require a high computational cost. This is because the diffusion process models the image formation process, but they may not be as effective as other models in capturing global features. Additionally, the high computational cost is a limitation that needs to be considered when using these models.

The current study aimed to answer the question: what is the impact of ViT on the Image Restoration domain? The study is based on a study of around 70 papers. First, every research work has been classified into the seven fields of image restoration. Then, a description of the state-of-the-art techniques in these seven fields is presented. Later, the advantages, drawbacks, and future research directions are discussed.

### 1.2. Related Surveys

To our knowledge, no literature survey has targeted this problematic. The existing surveys about ViT are more generic in scope and do not consider the image restoration domain’s peculiarities. For example, Khan et al. [18] have explored the use of vision transformers in popular recognition tasks, generative modeling, multi-modal tasks, video processing, low-level vision, and three-dimensional analysis. Han et al. [19] undertook a generic review of ViT for different computer vision tasks; they compared their advantages and drawbacks. Then, they classified the tasks into four classes: low-level vision, high/mid-level vision, backbone network, and video processing. They also discussed the application of vision transformers to real devices. Islam et al. [20] provided an overview of the best-performing modern vision Transformer methods and compared the strengths, weaknesses, and computational costs between vision Transformers and CNN methods on the most benchmark datasets. Shamshad et al. [21] were more specific and focused on one domain. They presented a comprehensive review of the application of vision transformers in medical imaging, including medical image segmentation, classification, detection, clinical report generation, synthesis, registration, reconstruction, and other tasks. They also discussed unresolved problems in the architectural designs of vision transformers.

Looking for surveys that focused only on image restoration tasks, no one has elaborated a study about ViT architectures in this regard. For example, Su et al. [22] generally tackled deep learning algorithms without focusing on ViT specifically. They presented a comparative study of deep learning techniques used in image restoration, including image super-resolution, dehazing, deblurring, and denoising. They also discussed the deep network structures involved in these tasks, including network architectures, skip connection or residual, and receptive field in autoencoder mechanisms. They also focused on presenting an effective network to eliminate errors caused by multi-tasking training in the super-resolution task. In Table 1, the related surveys to our research are described based on two main characteristics. The first is the algorithm studied (CNN and/or ViT). The second is the area of the survey. As concluded, a specific survey that studies the effect of ViT in the Image Restoration domain is still lacking in the current state of the art.

### 1.3. Main Contributions and Organization of the Survey

A gap is noted in these related surveys. A specific survey that analyses the impact of only ViT-based architectures on the image restoration domain is still needed, considering all of its peculiarities. The main contributions elaborated in this research are:

1. The study lists the most important ViT-based architectures introduced in the image restoration domain, classified by each of the seven subtasks: Image Super-Resolution, Image Denoising, General Image Enhancement, JPEG Compression Artifact Reduction, Image Deblurring, Removing Adverse Weather Conditions, and Image Dehazing.
2. It describes the impact of using ViT, its advantages, and drawbacks in relation to these image restoration tasks.
3. It compares the efficiency metrics, such as The Peak Signal-to-Noise Ratio (*PSNR*) and the Structural Similarity Index Metric (*SSIM*) of the ViT-based architectures, on the main benchmarks and datasets used in every task of image restoration.
4. It discusses the most critical challenges facing ViT in image restoration and presents some solutions and future work.

This study is organized as follows, Section 2 introduces the ViT architecture and presents its success keys, including self-attention, sequential feature encoding, and feature positioning. Section 3 details the evaluation metrics used to assess the performance of image restoration algorithms. These evaluation metrics will be used later for the whole paper, and their definitions are worth describing from the beginning. Section 4 describes the state of the art of ViT-based architectures in each of the seven image restoration tasks. Section 5 presents a discussion of the advantages, drawbacks, and the main challenges of ViT in image restoration. Finally, in Section 6, our work is concluded alongside a description of the next research problematics to be addressed in future works.

## 2. Vision Transformer Model

In this section, we introduce ViT and the most important principles upon which it is built, including structure, self-attention, multi-headed self-attention, and the mathematical background behind ViT. The ViT was introduced by [15] in 2020. The ViT is a deep neural network architecture for image recognition tasks based on the Transformer architecture initially developed for natural language processing tasks. The main idea behind ViT is to treat an image as a sequence of tokens (typically, image patches). Then, the transformer architecture is used to process this sequence. The transformer architecture, on which ViT is based, has been adapted to many tasks and is effective in many of them, such as image restoration and object detection [23,24].

**Figure 2:**
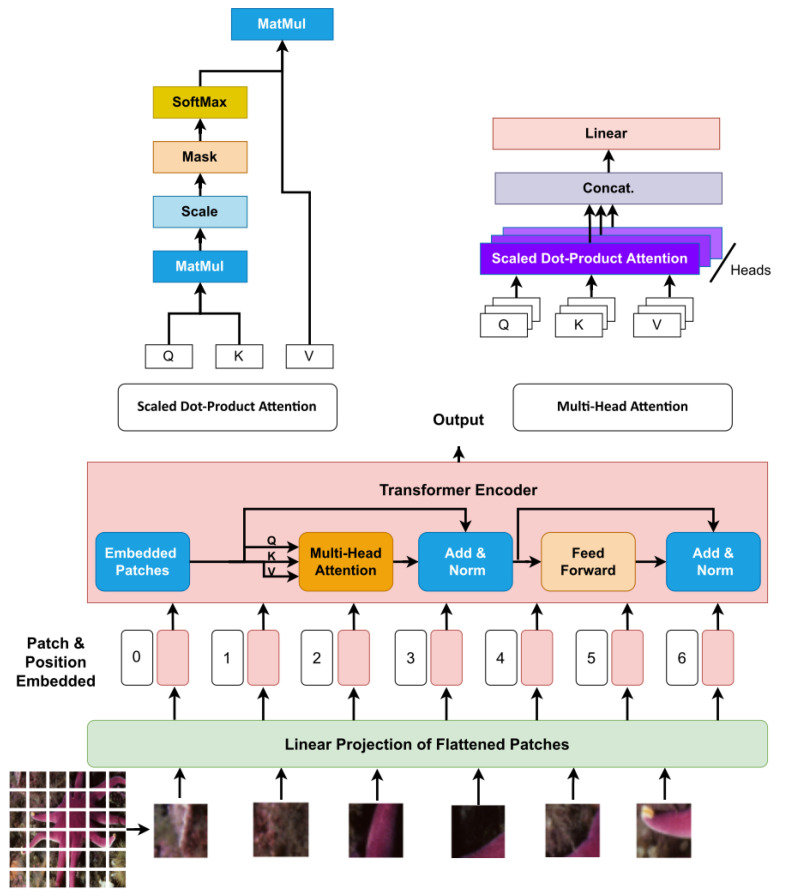
The general form of the ViT architecture.

The ViT is based on the Transformer architecture. Figure 2 shows the main stages of VIT architecture. where the image is tokenized and embedded by dividing it into a grid of non-overlapping patches, flattening each patch, and mapping it to a high-dimensional space through a linear transformation followed by normalization. This process, called tokenization and embedding, allows the model to learn both global and local information from an image.

The transformer architecture is designed to process any sequence, but it does not explicitly consider each token’s position in the sequence. To address this limitation, the ViT architecture uses pre-defined positional embeddings. These positional embeddings are additional vectors that encode the position of each token in the sequence, and they are added to the token embeddings before they are passed through the transformer layers. This mechanism allows the model to capture the relative position of the tokens and to extract spatial information from the image.

The core of the ViT architecture lies in the Multi-head Self-Attention (MSA). This mechanism allows the model to simultaneously attend to different parts of the image. It is composed of several different “heads”, each of which computes attention independently. These attention heads can attend to different regions of the image and produce different representations, which are then concatenated to form a final image representation. This allows the ViT to capture more complex relationships between the input elements, as it can attend to multiple parts of the input simultaneously. However, it also increases the complexity and computational cost of the model, as it requires more attention to heads and more processing to combine the outputs from all the heads. MSA can be expressed as follows:

(1) $$MultiHead\left(Q,K,V\right)=Concat\left(Head_{1},\;Head_{2},\ldots \;Head_{n}\right)$$

where $Head_{n}=self\;Attention\left(Q.W_{n}^{Q},\;K.W_{n}^{K},\;V.W_{n}^{V}\right)$, n is the number of heads. MSA depends on the self-attention mechanism introduced by the authors of [25]. The basic idea of self-attention is to estimate how closely one element relates to the other elements in the sequence of elements. In digital images, the image is divided into several patches, then each patch is converted into a sequence, and then self-attention estimates the relevance of one sequence to the rest of the sequences.

The self-attention mechanism is the backbone of transformers, which explicitly model the interactions and connections between all sequences of prediction tasks. The self-attention layer also collects global information and features from the entire input sequence, distinguishing it from CNN. It is for this reason that transformers have a larger model capacity. To understand more about the mechanism of self-attention, we may consider a sequence of 𝓃 elements $\left\{X_{1},\;X_{2},\;X_{3},\;\ldots X_{𝓃}\right\}$ by $X\in {\mathbb{R}}^{𝓃\times d}$, where d is the embedding dimension to represent each element. The purpose of the attention mechanism is to estimate the interactions and connections between all n elements by encoding each element and then capturing global information and features. In order to implement the attention mechanism, three learnable weight matrices must first be defined, including Queries $W^{Q}\in {\mathbb{R}}^{d\times d_{q}}$, Keys $W^{K}\in {\mathbb{R}}^{d\times d_{k}}$, and Values $W^{V}\in {\mathbb{R}}^{d\times d_{v}}$, where $d_{q}=d_{k}$. The X input sequence is projected by weights matrices $Q=X.W^{q}$, $K=X.W^{k}$, $V=X.W^{v}$. The output of attention layer Z is Equation (2):

(2) $$Z=softmax\left(\frac{QK^{T}}{\sqrt{d_{q}}}\right)V,$$

**Figure 3:**
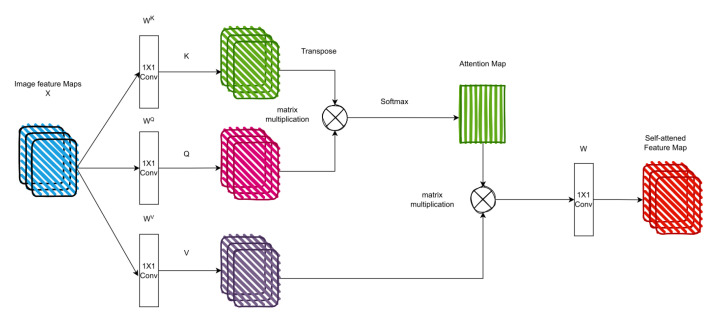
The self-attention model used in ViT.

The attention mechanism computes the dot product of *Q* (query) with all *K* (keys) for a given element in the sequence. Then the SoftMax operator is used for normalization to get the attention outputs. Each element in the sequence becomes the sum weight of all elements in the entire sequence; attention outputs generate these weights. Then, using the dot product, the attention output is multiplied by the *V* (value) matrix. Figure 3 shows an example of the self-attention model.

The main advantage of the self-attention mechanism compared to the convolution mechanism is that the values of the filters are calculated dynamically instead of static filters, as in the case of the convolution operation, where the filters remain the same for any input. In addition, self-attention is distinguished from standard convolution in the stability of permutations and changes in the number of insertion points. As a result, it can process irregular data and input. Based on literature reviews, it appears theoretically that the process of self-attention with positional encodings is more flexible in extracting local features than convolutional models [26]. Cordonnier et al. [27] investigated the connections between self-attention and convolution operations. Their results show that self-attention with sufficient parameters is a very flexible and more general process that can extract local and global features. Furthermore, based on previous studies and research findings on various computer vision tasks, self-attention can learn both local and global features. It also provides adaptive learning of kernel weights, as in research on deformable convolutions [28].

The ViT architecture requires a large amount of data for training to achieve optimal performance. This is a common challenge encountered with transformer models. To address this limitation, a two-stage training approach is often employed. In the first stage, supervised or self-supervised [24,29] pre-training is performed on a large dataset or a combination of several datasets [30]. In the second stage, the pre-trained weights are used to fine-tune the model on smaller or medium-sized datasets for specific tasks such as object detection, classification, and image restoration. This approach has been demonstrated to be effective in previous research, where it was shown that the accuracy of the Vision Transformer model decreased by 13% when trained only on the ImageNet dataset and increased when trained on the JFT dataset containing 300 million images. This highlights the importance of pre-training transformer models for vision and language on large-scale datasets.

Obtaining labeled datasets for training AI models can be a significant challenge due to the high cost and difficulties associated with the process. To address this, self-supervised learning (SSL) has emerged as a promising alternative to traditional supervised learning. SSL enables training models on a large number of parameters, up to a trillion, as demonstrated in [31]. The basic principle of SSL is to train a model on unlabeled datasets by applying various transformations to the images, such as slight adversarial changes or replacing one object with another in the same scene, without altering the semantics of the base class. This allows the model to learn from the data without needing manual labeling. For more information and a comprehensive survey of SSL technology, readers can refer to references [32,33].

## 3. Evaluation Metrics

This section introduces the most widely used image quality measurement methods in image restoration. In addition, we present the most modern methods for measuring image quality based on ViT. The quality of digital images can be defined based on the measurement method. Measurement methods depend on viewers’ perceptions and visual attributes. Image Quality Assessment (IQA) methods are classified into subjective and objective methods [34]. Subjective methods rely on image quality evaluators, but these methods take a lot of time, effort, and cost due to large datasets. Therefore, objective methods are more appropriate in image restoration tasks, according to [35]. Objective methods need the ground-truth image and the predicted image. *PSNR* is one of the most widely used image quality measures. *PSNR* is maximum signal to maximum noise. In image restoration, the *PSNR* results from divisibility between the maximum pixel value and Mean Square Error (*MSE*) between the ground-truth image and the predicted image. The *PSNR* is defined as:

(3) $$PSNR\left(i_{gt},\;i_{p}\right)=10\;\log_{10}\left(\frac{max^{2}}{MSE}\right),$$

where max is the maximum pixel value, i.e., the max value of 255 for 8-bit color depth and MSE is defined as:

(4) $$MSE\left(i_{gt},\;i_{p}\right)=\frac{1\;}{t}\sum_{k=1}^{t}{\left(i_{gt}\left(k\right)-i_{p}\left(k\right)\right)}^{2},$$

where $i_{gt}$ is the ground truth image, and $i_{p}\;$ is the predicted image. The *PSNR* approach can be misleading because the predicted image may not be perceptually similar to a ground truth image [36,37]. However, the *PSNR* approach is still used in all image restoration research, and research results are also compared using it. Human perception is the best measure of image quality for its ability to extract structural information [38]. However, ensuring human visual validation of the data remains very expensive, especially with large datasets. An image quality metric that measures the structural similarity between ground truth images and predicted images by comparing luminance, contrast, and structural information is called *SSIM* [39].

(5) $$SSIM\left(i_{gt},\;i_{p}\right)=\frac{\left(2\mu_{i_{gt}}\mu_{i_{p}}+{𝒸}_{1}\right)\left(2\sigma_{i_{gt}i_{p}}+{𝒸}_{2}\right)}{\left(\mu_{i_{gt}}^{2}+\mu_{i_{p}}^{2}+{𝒸}_{1}\right)\left(\sigma_{i_{gt}}^{2}+\sigma_{i_{p}}^{2}+{𝒸}_{2}\right)},$$

where $\mu_{i_{gt}}$ is the average value for the ground truth image, $\mu_{i_{p}}$ is the average value of the predicted image, $\sigma_{i_{gt}}$ is the standard deviation for the ground truth image, $\sigma_{i_{p}}$ is the standard deviation of the predicted image, and $\sigma_{i_{gt}i_{p}}=\mu_{i_{gt}i_{p}}–\;\mu_{i_{gt}}\mu_{i_{p}}$ is the co-variation. $c_{2}\;is\;{\left(k_{2}L\right)}^{2},\;\mathrm{and}\;c_{1}\;is\;{\left(k_{1}L\right)}^{2}$ are two variables to avoid division by zero, $k_{2}=0.03,$ and $k_{1}=0.01$.

In image restoration research, two of the most widely used image quality metrics are *SSIM* and *PSNR*. *SSIM*, in particular, is based on the human perception of structural information in images and is commonly used for generic images, as discussed in [40]. However, when applied to medical images, where brightness and contrast may not be consistent, the *SSIM* metric can be unstable, as reported in [41]. To address this issue, the authors in [42] proposed a model for measuring the perceptual quality of images. They tested this model using the Restormer model [43] in various noise removal and adverse weather conditions. They then applied an object detection model to the same images before and after using the Restormer model. They found that while *SSIM* and *PSNR* results are often unstable, the results from the object detection model were more consistent. This led the authors to propose a Grad-CAM model to measure image quality, which bridges the gap between human perception and machine evaluation [44].

Recently, Cheon et al. [45] proposed a new Image Quality Transformer (IQT) which utilizes the transformer architecture. IQT extracts perceptual features from both ground-truth and predicted images using a CNN backbone. These features are then fed to the transformer encoder and decoder, which compares the ground-truth images and the predicted images. The transformer’s output is then passed to a prediction head, which predicts the quality of the images. In further research, Conde et al. [46] replaced the IQT encoder-decoder with the Conformer architecture, which utilizes convolution layers from the Inception-ResNet-v2 model [47] along with attention operations to extract both global and local features.

## 4. Image Restoration Tasks

**Figure 4:**
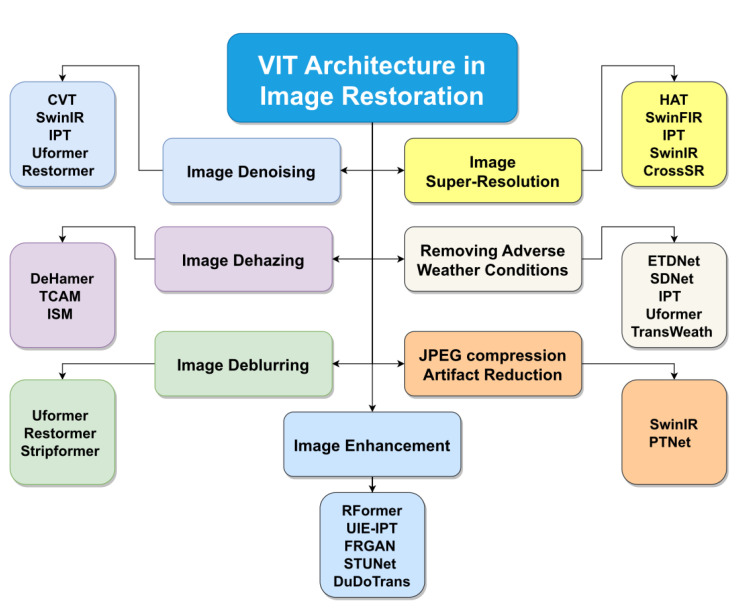
The most popular image restoration techniques based on ViT.

In this section, we will introduce all transformer-based image restoration tasks, in addition to presenting a comparative study between all models of vision transformers in each sub-task. Figure 4 presents a taxonomy of all vision tasks with the most common head transformer models.

### 4.1. Image Super-Resolution

**Figure 5:**
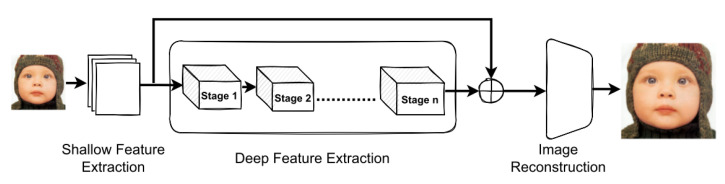
The block diagram of the best ViT-based super-resolution models.

Super-resolution is a technique for reconstructing high-resolution images from low-resolution input images. SR has been widely applied in image restoration tasks due to its value in various applications and its ability to overcome the limitations imposed by imaging systems [48]. SR can be broadly classified into two categories: multi-image super-resolution (MISR) and single-image super-resolution (SISR). MISR generates a single high-resolution image from multiple low-resolution images, while SISR generates a high-resolution image from a single low-resolution input. Vision transformer (VT)-based SR models, such as HAT [49], SwinIR [50], and SwinFIR [51], have gained widespread adoption in image restoration and are depicted in Figure 5 as a general block diagram.

In most applications, obtaining multiple images of the same scene is often complex and costly. Therefore, using only a single image in super-resolution technology is a suitable and effective solution for many applications. In most research papers, low-resolution images are typically obtained by applying a degradation model to high-resolution images, which involves blurring followed by downsampling. Traditional methods use interpolation and blur to enhance low-resolution images and rely on iterative optimization frameworks. Yang et al. [52] conducted an investigative comparison of ten high-resolution papers that utilize iterative optimization techniques and sparse representation, including [53,54,55].

Traditional methods for image restoration are often ineffective and time-consuming. In contrast, deep neural networks can learn from large amounts of training data rather than relying on complex statistical models. With the advancement of computing devices and GPUs, neural networks have become more prevalent. In recent years, the vision transformer has emerged as a strong contender in the field of computer vision, including in the area of image SR. According to the results of many research papers, vision transformers have shown high accuracy and efficiency in SR techniques. In this survey, we can classify super-resolution transformer networks based on the input size, the type of images, and the type of task. A low-resolution image $\hat{X}\;$ can be expressed as

(6) $$\hat{X}=D\left(X⊗B\right)↓+N,$$

where X is a high-resolution image, B is effect blur, and N is random noise. SR models based on transformers can be divided based on the nature of the images. Using a generic image to train super-resolution models is the most common and comprehensive method in most super-resolution applications. One of the most famous models is SwinIR [50], which is widely popular. SwinIR is an image restoration model that does more than one task depending on the type of training. SwinIR is inspired by the swin v1 [56] transformer used in image classification. SwinIR consists of a convolutional layer at the beginning called shallow feature extraction. In the middle, SwinIR consists of 36 consecutive swin blocks divided into six stages; each stage consists of 6 blocks and is called deep feature extraction. Finally, the reconstruction layer, which contains more than one type, depending on the task, in super-resolution, is an enlargement layer for the image dimensions. Because of the high efficiency of the swin model in extracting global and local features, it has also been used in many super-resolution models such as SWCGAN [57], SwinFIR [51] and 3DT-Net [58].

The HAT [49] model is a model that combines self-attention and channel attention, so it has a high ability to aggregate features. HAT also proposed an overlapping cross-attention module to collect information through cross-windows in a new and highly efficient way. Based on the results shown in Table 2, Table 3 and Table 4, HAT is the highest model in *PSNR* and *SSIM* scores across all scales. The SwinFIR model is a recent variation of the SwinIR model that utilizes Fast Fourier Convolution (FFC) components to extract global information suitable for the task of super-resolution. This is achieved by combining global and local features extracted by the FFC and residual modules.

Recently, authors in [65] proposed the first transformer-based light field super-resolution (LFSR) processing model, called SA-LSA. The model divides each light field into a set of image sequences and utilizes a combination of convolutional layers and a self-attention network to reinforce non-local spatial angular dependencies in each sequence. Table 5 compares SA-LSA with other super-resolution models on light field images.

SWCGAN uses a swin-inspired switched window self-attention mechanism combined with the GAN model to combine the advantages of swin switches with convolutional layers. SWCGAN has been applied to remote-sensing images. Table 6 compares super-resolution models on remote sensing images.

Transformers have also been applied to the super-resolution of medical images, such as in the case of the ASFT model [68]. ASFT consists of three branches, two of which transmit similar features from MRI slices, and the third branch builds the super-resolution slice. The performance of ASFT is compared with other super-resolution models on MIR medical images in Table 7 of the corresponding paper.

The 3DT-Net architecture is a transformer-based approach that leverages the spatial information in the hyperspectral images. It utilizes multiple layers of the Swin Transformer in place of 2D-CNN layers and employs 3D convolutional layers to take advantage of the spatial spectrum of the data. This architecture has been applied to hyperspectral image processing, which inherently possesses multi-dimensional characteristics. It is effective in this task, and it is worth noting that the performance of 3DT-Net has been evaluated and compared against other state-of-the-art models on hyperspectral images, as shown in Table 8.

Table 9 presents a collection of various super-resolution models that do not belong to a standardized dataset. The authors in [73] propose a transformer-based super-resolution texture network, called TTSR. This model uses reference images to create a super-resolution image from multiple low-resolution images and reference images. The VGG model [74] extracts features from the reference images. Additionally, a self-attention-based transformer model is proposed for extracting and transferring texture features from reference and low-resolution images.

A transformer-based super-resolution architecture, called CTCNet, was proposed by Gao et al. [75] for the task of face image super-resolution. CTCNet is composed of an encoder-decoder architecture and a novel global-local feature extraction module that can extract fine-grained facial details from low-resolution images. The proposed architecture demonstrated promising results and potential for the task of face image super-resolution. BN-CSNT [76] is a network that combines channel splitting and the Swin transformer to extract context and spatial information. Additionally, it includes a feature fusion module with an attention mechanism, and it has been applied to thermal images to address the super-resolution problem. One of the main advantages of transformers in super-resolution is their adaptability to different types of images, similar to CNNs.

### 4.2. Image Denoising

Noise reduction, or denoising, is crucial in image processing and restoration. Removing noise from images is often used as a preprocessing step for many computer vision tasks, and it is also used to evaluate optimization methods and image prior models from a Bayesian perspective [77]. There are various approaches to reducing noise, including traditional methods such as BM3D [78], which enhances image contrast by assembling blocks of similar images from 3D images. There are also machine learning-based methods, and in [79], there is a comprehensive study on the use of machine learning for noise reduction in images. The wide range of noise reduction models is due to their widespread use in signal processing. In recent years, deep learning methods have been dominant in terms of perceptual efficiency and quality for noise reduction. For an overview of deep learning models based on CNNs for noise reduction, see [80]. In this section, we will focus on vision transformer-based noise reduction algorithms. Noisy images production can be expressed mathematically as

(7) y=x+n,

where y is a noisy image, x is a clean image, and n is the random noise. Noise removal algorithms based on deep learning are divided into two methods.

**Figure 6:**
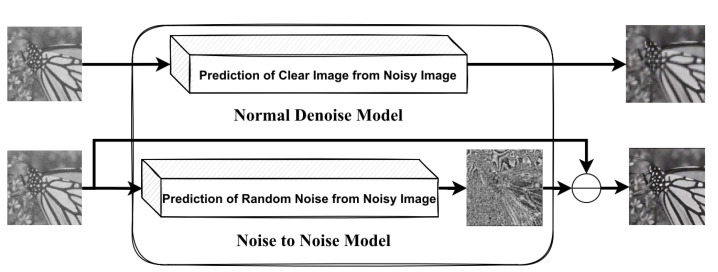
The main method for removing noise from images using transformer-based approaches. The top image shows a traditional technique that focuses on restoring clear image details from a noisy image. The bottom image depicts a more efficient method that estimates the random noise present in the noisy image and then subtracts this estimated noise from the image to produce a noise-free result.

There are two main approaches for removing noise from images using ViT, as shown in Figure 6. The first approach, known as “noise-to-noise,” involves using deep learning networks to estimate the noise in an image and then subtracting the estimated noise from the noisy image to generate a noise-free image. The second approach involves using deep learning networks to estimate noise-free images from noisy images directly. For example, Prayuda et al. [81] proposed a convolutional vision transformer (CVT) for noise removal using the noise-to-noise approach, which employs residual learning to reduce noise from the noisy image by estimating the relationship between the noisy image and its noise map. Several previous research studies, including SwinIR, Uformer, Restormer, and IPT, have used a single encoder for all image restoration tasks but have utilized multiple decoders or reconstruction layers depending on the specific task.

Liu et al. [82] have proposed a new network called DnT for unsupervised noise removal from images. DnT combines a CNN and Transformer to estimate clean images from noisy images, using a loss function that is measured by pairs of noisy independent images generated from the input images. This method, known as R2R [83], effectively removes noise from images. In addition, the authors of [84] propose an efficient model called TECDNet, which utilizes a transformer for data encoding and a convolutional network for the decoder. Additionally, a convolutional network is incorporated to reduce computational complexity. Another transformer-based model for noise removal is TC-Net, which was introduced by Xue et al. [85]. This network consists of extra skip-connections, window multi-head self-attention, a convolution-based forward network, and a deep residual shrinkage network. The components of TC-Net work together to integrate features between layers, reduce computational complexity, and extract local features. The swin transformer is also frequently used in various shapes and structures for noise removal in image restoration tasks due to its ability to effectively extract important features from input images and its adaptability to different structures. Table 10 presents a comparison of various ViT models for the task of denoising in both generic and medical images. It can be observed that when the level of noise is high, the performance of the models, as measured by the *SSIM/PSNR* metrics, decreases.

Fan et al. [87] have used a swin transformer as a basic block inside the layers of the U-net model, and it is called SUNet. Li et al. [88] collected a real dataset of noisy polarized color images to remove the disturbing noise and used a conventional camera to take the same images and use these images as ground truth. Then they proposed a hybrid transformer model based on the attention mechanism to remove the disturbing noise from the polarized color images, called Pocoformer. The authors in [89] have proposed a model to improve image quality and reduce noise in Low-Dose Computed Tomography (LDCT). Their proposed model, called TransCT analyzes distorted images into High-Frequency (HF) and Low-Frequency (LF) components. Several convolutional layers extract content and texture features from LF and texture features from HF. The extracted features are then fed into a modified transformer with three encoders and decoders blocks to obtain well-polished features. Finally, the refined features are combined to restore high-quality LDCT images. LDCT images are the most common in diagnosing diseases. However, LDCT suffers from loud noises more than normal CT images. Therefore, the authors of [90] have proposed a new convolution-free Token-to-Token (T2T) transformer model to remove noise from LDCT images. Luthra et al. [91] have proposed a transformer that improves edge-to-edge using a Sobel filter called Eformer. In addition, non-overlapping self-attention based on a shifted window is used to reduce computational costs while using residual learning to remove natural noise in LDCT images.

### 4.3. Image Deblurring

**Figure 7:**
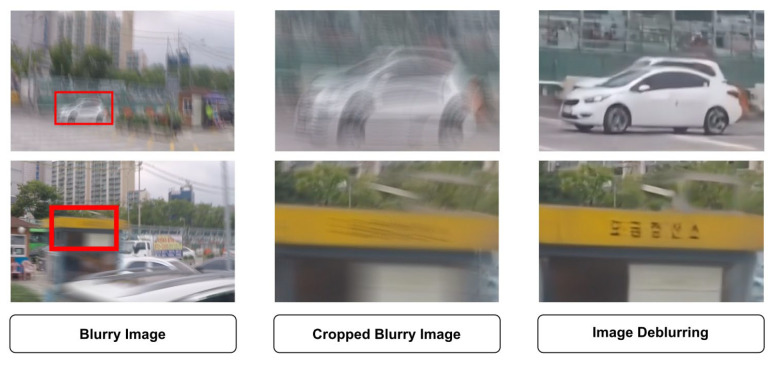
Some examples of blurry images processing.

Blurry images can be caused by various factors, such as the random movement of objects or the movement of the camera itself and physical limitations. This makes the problem of blurry images challenging to describe and solve. Blurry images are common in many image capture devices due to factors such as camera movement or scene changes [92]. It is not feasible to develop a mathematical formula that accurately captures the blur in an image, as it depends on a range of variables. In the past, various approaches have been proposed for deblurring images, including traditional methods. Wang et al. [93] made a comprehensive review of the traditional methods that occurred in common imaging and divided them into five frames according to the characteristics of each of them. Lay et al. [94] evaluated 13 single-image deblurring algorithms. Zhang et al. [95] presented a recent study on deep learning and CNN methods in terms of structure, loss function, and various applications. This paper will focus on transformer-based deep learning models to treat blurry images. Figure 7 presents some examples of blurry image processing. The leftmost image is a full, blurry image, the middle image is a cropped version of the blurry image, and the rightmost image is the reconstruction of the blurry image using the model.

Wang et al. [86] introduced a new transformer-based network called Uformer, consisting of a hierarchical U-net architecture. Uformer is based on two main designs: The locally enhanced window (LeWin) block significantly reduces computational cost when using it with high-resolution feature maps rather than window-based self-attention. Secondly, they designed a learnable multi-scale restoration modulator to adjust the spatial features at the multi-layer level. Zamir et al. [43] introduced a novel transformer called Restormer Transformer (Restormer). Transformers are characterized by high image quality accuracy but suffer from significant computational complexity. Restormer is a practical image restoration transformer that models global features and can be applied to large images. Restormer consists of a multi-Dconv head ‘transposed’ attention (MDTA) block. MDTA is distinct from the vanilla multi-headed [25] in calculating the inter-channel covariance to obtain an optimal attention map. An advantage of MDTA is that it uses global relationships of pixels and optimizes the local context for feature mapping. Table 11 illustrates various image deblurring models that are based on the ViT architecture. It is worth noting that all the datasets used in this demonstration are real-world data and not synthetic.

In order to reduce the computational complexity of transformers, the authors of [96] proposed a deep network called Multi-scale Cubic-Mixer. Besides, to reduce computational costs, they did not use any self-attention mechanism. The proposed model uses the fast Fourier transform to calculate the Fourier coefficients to use the real and imaginary components and thus obtain the image without blurring. The proposed new network extracts the long-range and local features from blurred images in the frequency domain. The authors [97] have proposed a hybrid transformer called Stripformer, which relies on the attention of inter-strip and intra-strip [98] and which estimates motion blur from a blurred image by projecting motion blur in vertical and horizontal directions in the Cartesian coordinate. In addition, the vertical and horizontal features extracted at each pixel are stored to provide more information for subsequent layers in order to deduce the blur pattern. This method enables the sequential extraction of multi-scale local features. Therefore, the stripformer can remove long- and short-range blurred artifacts.

### 4.4. Image Dehazing

**Figure 8:**
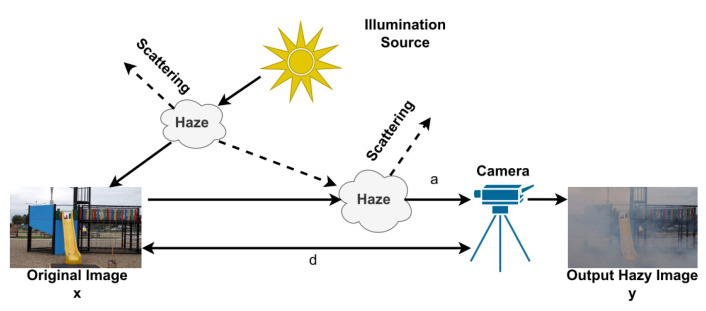
The atmospheric dispersion model by which the haze formation process is described.

Image dehazing involves the removal of non-linear noise produced by a turbid medium, such as dust and fog, which impairs the visibility of images and objects. The presence of hazing in images adds complex and difficult-to-predict noise, making it a challenging problem in image restoration. In addition, hazing reduces the accuracy and efficiency of computer vision algorithms. The generation of hazy images can be described using the atmospheric scattering model:

(8) $$y=x*e^{-\beta 𝒹}+a\left(1-e^{-\beta 𝒹}\right),$$

where y is a hazy image, x is an image without hazing, a is global atmospheric light, β is the scattering coefficient, and 𝒹 is the distance between the camera and the object. Many computer vision algorithms degrade in hazing scenes. Figure 8 describes an atmospheric dispersion model describing the hazing formation. 

The RESIDE dataset is commonly used for training and testing models for dehazing images. It includes 13,990 images in the Indoor Training Set (ITS), 500 images in the Synthetic Object Testing Set (SOTS), and 20 images in the Hybrid Subjective Testing Set (HSTS). To address the inconsistency problem of various features from CNN and transformer models, the authors of [99] propose a feature modulation module for combining hierarchical global features from transformers and hierarchical local features from CNN for image dehazing. Furthermore, Gao et al. [100] proposed a model that combines residual and parallel convolutional networks with a transformer-based channel attention module for detailed feature extraction called TCAM. TCAM consists of a channel attention module and a spatial attention module, and the channel attention module improves features along the channel.

Table 12 presents a comparison of different ViT models for the task of image dehazing. It can be observed that many of these models utilize the RESIDE dataset, which consists of both indoor and outdoor real-world images, for their evaluation. Li et al. [101] proposed a new two-phase de-hazing network. The first phase is based on the swin transformer and extracts key features from blurred images. In addition, the swin architecture has been improved by adding an Inter-block Supervision Mechanism (ISM). In the second phase, convolutional layers are used to extract local features and are also merged. The authors [102] have proposed a dual-stream network consisting of a CNN and transformers to extract global and local features. In addition, they proposed an atmospheric light estimation model based on atmospheric veils and partial derivatives. Song et al. [103] introduced a DehazeFormer model for removing hazing from generic images. Besides, they use multi-head self-attention (MHSA) that is dynamically trainable to adapt to different generics of images. Additionally, they suggest a soft reconstruction module based on SoftReLU. The contributions of Song et al. are to propose a modified normalization layer, spatial feature aggregation system, and soft activation function. Zhao et al. [104] suggest an improved framework for complementary features that relies on forcing the model to learn weak complementary features rather than iteratively learning ineffective features of the model. In addition, they proposed the Complementary Feature Selection Module (CFSM) to identify the most valuable features in learning.

### 4.5. Image JPEG Compression Artifact Reduction

With the significant development in cameras, most devices have become highly dependent on images, including airplanes, cars, and smartphones. Images and videos require huge storage space and high internet speed. This leads to the need for image compression and storage systems in all applications. However, most compression methods result in compression noises, such as compression artifacts [105]. JPEG is the most popular image compression technology on the Internet. JPEG compression technology consists of four stages: discrete cosine transformation (DCT), entropy coding, block division, and quantization. JPEG suffers from image dropouts at spatial boundaries because JPEG ignores the spatial connections between image blocks. Therefore, there are approaches based on filters [106,107,108] to improve the quality of images after compression, but they suffer from image blur. Deep learning techniques have emerged in the past few years to solve the JPEG compression artifact problem by mapping compressed images to ground-truth images [109,110,111]. In Table 13, a comparison of transformer-based models for removing JPEG compression artifacts is presented. The evaluation is done in terms of quality, the number of parameters, and the datasets used for a single image and the pairs of stereo images.

Modern techniques are divided into two parts in JPEG compression artifact reduction; the first is using one image to solve the problem, and the second is using two or more images. Each method has advantages and disadvantages. In this work, we present the latest JPEG compression artifact reduction techniques based on transformers. The most famous transformer-based model is SwinIR [50] which was mentioned before. Transformers are distinguished in capturing global and local features from CNN, which only extracts local features. SwinIR is a model that addresses more than one problem in image restoration, and the last layers of it are changed to suit the solution of each problem. A single convolution layer is used in the last block of the SwinIR model to remove the JPEG compression artifact. In addition, Charbonnier loss [113] was used in training the SwinIR model. The second type of JPEG artifact removal is the use of stereo image pairs. 

### 4.6. Removing Adverse Weather Conditions

**Figure 9:**
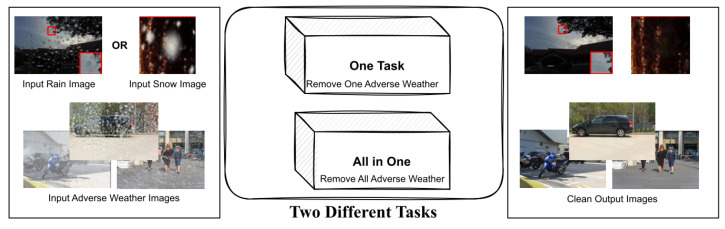
Illustration of the two common types of adverse weather removal algorithms. The top image shows a technique that removes a single type of adverse weather, such as rain or snow. The bottom image depicts a more efficient approach that is capable of removing multiple types of adverse weather at the same time.

Many computer vision algorithms, including those used for detection, depth estimation, and segmentation [114,115,116], are sensitive to the surrounding conditions and can be affected by factors such as adverse weather. These algorithms have important applications in various systems that are integral to our daily lives, such as navigation and surveillance systems and Unmanned Aerial Vehicles (UAVs) [117,118,119]. As a result, it is necessary to address hostile weather conditions such as fog, rain, and snow that can negatively impact the reliability of vision systems. Traditional methods for removing adverse weather are often based on empirical observations [120,121,122]. However, these methods must be tailored to each weather condition, as an all-weather method is generally ineffective. Additionally, there are algorithms based on CNNs that have been developed for removing adverse weather, including deraining [123,124,125,126], desnowing [127,128,129], and raindrop removal [130,131]. In Figure 9, it can be observed that there are several transformer-based approaches for removing adverse weather conditions, which can be broadly categorized into two groups: methods that focus on removing a specific type of adverse weather and methods that are capable of removing a variety of adverse weather conditions.

Qin et al. [132] proposed ETDNet, which utilizes transformers to extract features from individual images to remove rain streaks. ETDNet employs expansion filters at different rates to estimate the appropriate kernel for rainy images, thereby allowing for the extraction of features in an approximate to exact manner. The authors of [133] utilized a Swin-transformer [56] for rain removal from single images, utilizing a Swin block in three parallel branches referred to as Mswt. Deep and shallow features are extracted through the use of four consecutive Mswt and skip-connections between Mswt. The authors [134] proposed a two-stage training and fine-tuning approach called Task-Agnostic Pre-Embedding (TAPE). TAPE is trained on natural images, and then the knowledge is used to help remove rain, snow, and noise from images in a single model. The authors [135] proposed a model that eliminates adverse weather conditions at once called TransWeather, which consists of a single decoder. The weather-type queries are identified by a multi-headed attention mechanism and matched with the values and keys taken from the features extracted from the transformer encoder. To reconstruct the images after identifying the type of deterioration, hierarchical features from the encoder and features from the decoder are combined and then projected by a convolutional block on the image space. Therefore, TransWeather consists of one encoder and one decoder to eliminate adverse weather and generate a pure image. Transformers can extract important global features compared to CNN. However, when patches are large, transformers fail to pay attention to small information and detail. 

Table 14 presents a comparison of transformer-based models for removing adverse weather conditions. The models can be divided into two categories: those that remove a specific type of adverse weather and those that are capable of removing multiple types of adverse weather. It is important to note that it is generally preferable to use models that can handle multiple types of adverse weather conditions in real-world applications. For example, Liu et al. [136] designed an image restoration model called SiamTrans, inspired by Siamese transformers. SiamTrans is trained on an extensive dataset in a noise reduction task. Then knowledge transfer is used to train SiamTrans on many low-level image restoration tasks, including demoireing, deraining, and desnowing. In addition, the SamTrans consists of the encoder, decoder, self-attention units, and temporal-attention units. Two Uformer and Restormer models were previously introduced in detail in the image deblurring section.

### 4.7. General Image Enhancement

**Figure 10:**
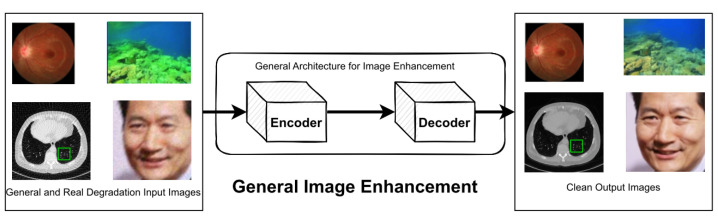
Illustration for general image enhancement, which does not fall under any specific sub-task of the image restoration.

Image restoration is a technique used to restore missing information in images and has been applied to various tasks such as super-resolution, denoising, deblurring, dehazing, JPEG compression artifact reduction, and the removal of adverse weather conditions. However, some research involving image datasets may not fit neatly into any specific image restoration task. These datasets may take the form of images captured of different qualities or images that have undergone significant degradation due to natural or artificial factors, as shown in Figure 10. The datasets that need improvement have two forms. The first form is images taken twice, once with high quality and once with low quality. The second form is images suffering from a lot of image degradation, including noise, downsample, blur, hazing, and adverse weather conditions.

Deng et al. [137] have created a clinical dataset (real fundus). This dataset consists of 120 real pairs of low-resolution and high-resolution images. They also proposed a new transformer-based GAN called RFormer. RFormer is a true degradation restoration model for fundus images. RFormer consists of a generator and a discriminator. The generator is based on the U-Net architecture and is mainly based on the Window-based Self-Attention Block (WSAB) but has been replaced by the Multilayer Perceptual (MLP) with the Feed-Forward Network (FFN). The WSAB generates more realistic images that capture long-range dependencies and non-local self-similarities. The discriminator model relies on a transformer to distinguish between real and fake images to monitor the quality of the generator. The discriminator is based on PatchGAN [138], which has a final layer as N × N × 1.

Boudiaf et al. [139] have employed an Image Processing Transformer (IPT) model [59] to address the problem of noise and distortion in underwater images. The IPT model was trained on a large number of images for tasks such as super-resolution and noise removal, and it has shown promising results in this context. In a separate study, the authors in [140] focused on restoring and improving degraded facial images, due to their significance in various applications. They proposed the FRGAN model, which can restore facial features and enhance accuracy. The FRGAN comprises three stages: a head position estimation network, a Postural Transformer Network, and a Face Generative Adversarial Network. Additionally, a new loss function called Swish-X was also proposed for use in the FRGAN model. Souibgui et al. [141] presented a flexible document image enhancement model called DocEnTr, based on transformers. DocEnTr is the first encoder-decoder-based image document restoration model that uses transformers without any CNNs. The encoder extracts the most salient features from the positional pixels and converts them into encoded patches, without the use of convolution layers, while the decoder reconstructs the images from the encoded patches. Table 15 presents the results of various ViT models in enhancing the quality of images. It is important to note the diversity of the datasets used in these evaluations, which include scenarios that do not require mathematical degradation models.

Puyang et al. [142] have created two datasets for blind face restoration, known as BFR128 and BFR512. They also present a comprehensive model for facial image degradation, which includes noise, down-sampling, blur, and JPEG compression artifacts, and combine them in a complete degradation scenario. In addition, they propose the U-net model and replace the down-sampling and up-sampling layers with swin-transformer blocks. In [143], they propose the Sinogram Restoration Transformer (SRT) model, which is inspired by the swin transformer and long-range dependency modeling. They also introduce the Residual Image Reconstruction Module (RIRM) for restoring X-ray sinograms and a differentiable DuDo Consistency Layer to preserve consistency in the sinograms, leading to the creation of high-quality Computed Tomography (CT) images. In [144], the authors propose the U2-Former transformer based on a nested U-shaped architecture to facilitate feature transfer between different layers. The model utilizes two overlapping or nested structures: an inner U-shape built on the self-attention block and an external U-shape to create a deep encoding and decoding space. This model is complex, but its strength lies in its large transformer depth, leading to high efficiency in image restoration tasks such as reflection removal, rain streak removal, and dehazing.

Yan et al. [145] propose a medical MRI model called SMIR, which combines the advantages of residual contact with those of the transformer. SMIR consists of a feature extraction unit and a reconstruction unit, with the Swin transformer used as the feature extractor. The proposed model is applied to different ratio radial filling trajectory samples. The authors in [146] proposed a U-net-based transformer to combine the advantages of convolutional layers and transformers. They used several convolution layers in the encoder, the encoder vector, the transformer encoder block, and the same structure in the decoder. In addition, they used self-learning and contrastive loss.

## 5. Discussion and Future Work

In this section, we present the advantages and limitations of ViT in image restoration as well as the new possible research directions arising from these limitations. The current study has analyzed the use of transformer models, specifically the ViT, in the field of image restoration. Our review of the literature found that ViT has been widely applied to various tasks within image restoration, including Super-Resolution, Denoising, General Image Enhancement, JPEG Compression Artifact Reduction, Image Deblurring, Removing Adverse Weather Conditions and Image Dehazing. It has generally achieved strong results compared to traditional methods and other deep learning models such as CNNs and GANs.

**Figure 11:**
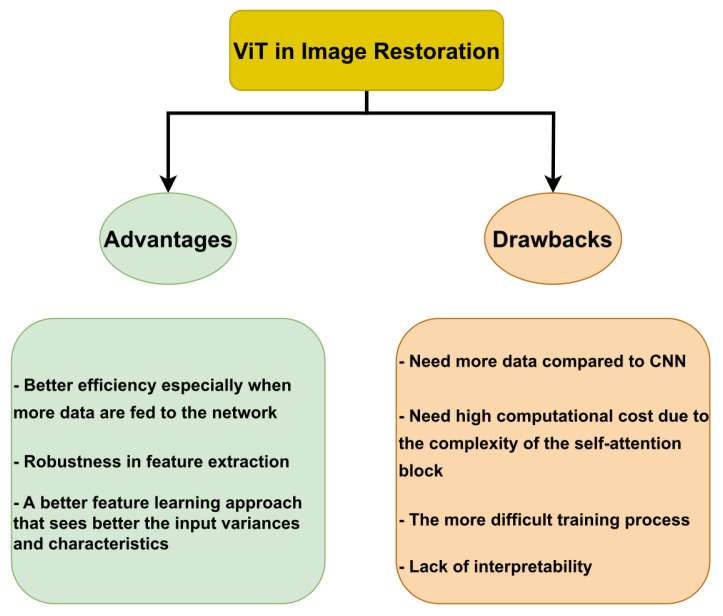
The main advantages and drawbacks of ViT in Image Restoration.

One of the key advantages of using ViT in image restoration is its ability to extract global features and its stability during training. This allows ViT to effectively model complex relationships within the data and generalize well to new examples. In addition, ViT has been shown to be flexible and perform well on various types of data, including generic images, medical images, and remote sensing data. However, there are also some limitations to using ViT in image restoration. One major issue is the high computational cost of training ViT models, which can make them impractical for some applications. However, ViT performs better than CNN models when trained on large datasets. The performance of different versions of ViTs in the task of image classification shows that its performance increases while increasing the number of training images compared to the CNN-based ResNet (BiT) model [15]. In contrast, ViT suffers from slow training (large training time) due to the self-attention process, which requires higher computational costs than CNN models [15]. In addition, ViT is less effective at extracting local features and may struggle to capture close-range dependencies within the data. This can be a drawback in tasks requiring detailed, fine-grained image analysis. Figure 11 summarizes the advantages and drawbacks of ViT in image restoration.

Despite these limitations, our review suggests that ViT has strong potential for use in image restoration and has already demonstrated impressive results in a variety of tasks. However, further research is needed to address the challenges of using ViT in image restoration, such as reducing the computational cost and improving its ability to extract local features. This could involve developing more efficient self-attention mechanisms, adapting the model to the specific characteristics of image restoration tasks, or exploring hybrid approaches that combine both ViT and other deep learning models.

Several directions for future research could help to further advance the use of ViT in image restoration. One potential avenue is to explore ways to reduce the computational cost of training ViT models, such as using more efficient self-attention mechanisms or adapting the model to the specific characteristics of image restoration tasks. Another area for investigation is the development of more interpretable ViT models, which could help better understand and explain the model’s decisions during the restoration process. This could involve incorporating additional interpretability mechanisms into the model architecture or designing more transparent loss functions. In addition, further research could focus on developing hybrid approaches that combine the strengths of ViT with other deep learning models, such as CNNs. This could allow for the use of the best features of both models in a single image restoration pipeline, potentially resulting in improved performance.

## 6. Conclusions

In conclusion, ViT models have gained widespread attention in computer vision. They have demonstrated superior performance to traditional models such as CNNs in various tasks, including image classification, object detection, action recognition, segmentation, and image restoration. This survey has explicitly focused on the use of ViT models, in the field of image restoration. We have reviewed a range of approaches based on vision transformers, including image super-resolution, denoising, enhancement, JPEG compression artifact reduction, deblurring, removal of adverse weather conditions, and dehazing. We have also presented a comparative study of the state-of-the-art vision transformer models in each of these tasks and have discussed the main strengths and drawbacks of their adoption in image restoration. Overall, our review suggests that vision transformers offer a promising approach to addressing the challenges of image restoration and have demonstrated strong performance in many tasks. However, there are also limitations to their use, including a high computational cost and difficulty in extracting local features. To further advance the use of vision transformers in image restoration, it will be important for future research to address these limitations and explore ways to improve the model’s performance and efficiency.

## Figures and Tables

**Table 1:** Comparison between our survey and the related surveys in image restoration.

Method	Year	Algorithm	Region of Interest
[18]	2022	ViT	Generative modeling, low-level vision (e.g., image super-resolution, image enhancement, and colorization), Recognition tasks (e.g., image classification, object detection, action recognition, and segmentation), video processing (e.g., activity recognition, video forecasting), multi-modal tasks (e.g., visual question answering, visual reasoning, and visual grounding), and three-dimensional analysis (e.g., point cloud classification and segmentation)
[19]	2022	ViT	Backbone network (e.g., supervised pretraining and self-supervised pretraining), high/mid-level vision (e.g., object detection, segmentation, and pose estimation), low-level vision (e.g., image generation, and image enhancement), video processing (e.g., video inpainting, and video captioning), multimodality tasks (e.g., classification, image generation, and multi-task), and efficient transformer (e.g., decomposition, distillation, quantization, and architecture design)
[20]	2022	ViT, CNN	Fundamental concepts, a background of the self-attention mechanism, strengths and weaknesses, computational cost, and comparison of ViT and CNN performance on benchmark datasets
[21]	2022	ViT	Medical image segmentation, detection, classification, reconstruction, synthesis, registration, and clinical report generation
[22]	2022	CNN, ViT	General deep learning techniques in super-resolution, dehazing, deblurring, and image denoising. Then, a summary of the leading architectural components involved in these tasks, such as residual or skip connection, receptive field, and unsupervised autoencoder mechanisms.
Our	-	ViT	Seven image restoration tasks are deeply studied: image super-resolution, image denoising, general image enhancement, JPEG compression artifact reduction, image deblurring, removing adverse weather conditions, and image dehazing.

**Table 2:** Generic Image Super-Resolution-Based on ViT for scale ×2. Best records are emphasized in bold font.

Method	Training Dataset	Set14 *PSNR*/*SSIM*	BSD100 *PSNR*/*SSIM*	Manga109 *PSNR*/*SSIM*	Parameters
HAT [49], 2022	DF2K	**35.29/0.9293**	**32.74/0.9066**	**41.01/0.9831**	20.8M
SwinFIR [51], 2022	DF2K	34.93/0.9276	32.64/0.9054	40.61/0.9816	13.99M
IPT [59], 2021	ImageNet	34.43	32.48	-	46.0M
SwinIR [50], 2021	DF2K	34.61/0.9260	32.55/0.9043	40.02/0.9800	**11.8M**
CrossSR [60], 2022	DIV2K	33.99/0.9218	32.27/0.9000	-	18.3M
SRT [61], 2022	DIV2K	33.95/0.9207	32.35/0.9018	-	11M
ELAN [62], 2022	DIV2K	33.94/0.9207	32.30/0.9012	39.11/0.9782	582K
HIPA [63], 2022	DIV2K	34.25/0.9235	32.48/0.9040	39.75/0.9794	19.2M
ACT [64], 2022	ImageNet	34.68/0.9260	32.60/0.9052	40.11/0.9807	46.0M

**Table 3:** Generic Image Super-Resolution-Based on ViT for scale ×3. Best records are emphasized in bold font.

Method	Training Dataset	Set14 *PSNR*/*SSIM*	BSD100 *PSNR/SSIM*	Manga109 *PSNR/SSIM*	Parameters
HAT [49], 2022	**DF2K**	**31.47/0.8584**	**29.63/0.8191**	**36.02/0.9576**	20.8M
SwinFIR [51], 2022	DF2K	31.24/0.8566	29.55/0.8169	35.77/0.9563	13.99M
IPT [59], 2021	ImageNet	30.85	29.38	-	46.0M
SwinIR [50], 2021	DF2K	31.00/0.8542	29.49/0.8150	35.28/0.9543	**11.8M**
CrossSR [60], 2022	DIV2K	30.43/0.8433	29.15/0.8063	33.95/0.9455	0.68M
SRT [61], 2022	DIV2K	30.53/0.8460	29.21/0.8082	-	18.3M
ELAN [62], 2022	DIV2K	30.55/0.8463	29.21/0.8081	34.00/0.9478	590K
HIPA [63], 2022	DIV2K	30.38/0.8417	29.13/0.8061	33.82/0.9460	365K
ACT [64], 2022	DIV2K	30.80/0.8504	29.45/0.8127	34.86/0.9521	19.2M
HAT [49], 2022	ImageNet	31.17/0.8549	29.55/0.8171	35.47/0.9548	46.0M

**Table 4:** Generic Image Super-Resolution-Based on ViT for scale ×4. Best records are emphasized in bold font.

Method	Training Dataset	Set14 *PSNR/SSIM*	BSD100 *PSNR/SSIM*	Manga109 *PSNR/SSIM*	Parameters
HAT [49], 2022	DF2K	**29.47/0.8015**	**28.09/0.7551**	**33.09/0.9335**	20.8M
SwinFIR [51], 2022	DF2K	29.36/0.7993	28.03/0.7520	32.83/0.9314	13.99M
IPT [59], 2021	ImageNet	29.01	27.82	-	46.0M
SwinIR [50], 2021	DF2K	29.15/0.7958	27.95/0.7494	32.22/0.9273	**11.8M**
CrossSR [60], 2022	DIV2K	28.11/0.7842	27.54/0.7464	30.09/0.9077	-
SRT [61], 2022	DIV2K	28.69/0.7833	27.69/0.7379	30.75/0.9100	0.68M
ELAN [62], 2022	DIV2K	28.79/0.7856	27.70/0.7405	32.46/0.8975	18.3M
HIPA [63], 2022	DIV2K	28.87/0.7880	27.75/0.7429	-	18.3M
ACT [64], 2022	DIV2K	28.78/0.7858	27.69/0.7406	30.92/0.9150	601K
HAT [49], 2022	DIV2K	28.68/0.7832	27.62/0.7382	30.76/0.9111	365K
SwinFIR [51], 2022	DIV2K	29.02/0.7945	27.94/0.7463	31.77/0.9231	19.2M
IPT [59], 2021	ImageNet	29.27/0.7968	28.00/0.7516	32.44/0.9282	46.0M

**Table 5:** Light Field Super-Resolution (LFSR). The best records are in bold font.

Method	Scale	EPFL *PSNR/SSIM*	HCInew *PSNR/SSIM*	HCIold *PSNR/SSIM*	INRIA *PSNR/SSIM*	Parameters
SA-LSA [65], 2022	×2	34.48/0.9759	37.35/0.9770	44.31/0.9943	36.40/0.9843	3.78M
LFT-transformer [66], 2022		**34.80/0.978**	**37.84/0.979**	**44.52/0.995**	**36.59/0.986**	**1.16M**
SA-LSA [65], 2022	×4	28.93/0.9167	31.19/0.9186	37.39/0.9720	30.96/0.9502	3.78M
LFT-transformer [66], 2022		**29.25/0.921**	**31.46/0.922**	**37.63/0.974**	**31.20/0.952**	**1.16M**

**Table 6:** Image Super-Resolution based on ViT in the Remote Sensing field.

Method	Scale	Training Dataset	AID *PSNR/SSIM*	UCMerced *PSNR/ SSIM*	Parameters
TransENet [67], 2022	×2	UCMerced, AID	35.28/0.9374	34.03/0.9301	-
	×4		29.38/0.7909	27.77/0.7630	-
SWCGAN [57], 2022	×4		-	27.63	3.8M

**Table 7:** MRI Images Super-Resolution. The best records are in bold font.

Method	Scale	Training Dataset	Kirby21 *PSNR/SSIM*	ANVIL-adult *PSNR/SSIM*	MSSEG *PSNR/SSIM*	BraTS2018 *PSNR/SSIM*	Parameters
ASFT [68], 2022	×2	Kirby21	**43.68 ± 2.08/** **0.9965 ± 0.0014**	**40.96 ± 1.00/** **0.9906 ± 0.0013**	**41.22 ± 1.37/** **0.9978 ± 0.0004**	-	**1.85M**
CFTN [69], 2022			39.70 0.9847	-	-	-	21.93M
ASFT [68], 2022	×3		40.19 ± 2.04/0.9882 ± 0.0034	37.54 ± 1.10/0.9703 ± 0.0041	36.82 ± 1.43 /0.9868 ± 0.0021	-	1.85M
CFTN [69], 2022			36.03 0.9612	-	-	-	21.93M
Cohf-T [70], 2022	×3	-	-	-	-	34.84/0.9507	152M
	×4		-	-	-	33.26/0.9425	

**Table 8:** Hyperspectral Image Super-resolution ×4. Best records are in bold font.

Method	Training Dataset	Harvard *PSNR/SSIM*	CAVE *PSNR/SSIM*	Houston *PSNR/SSIM*	Pavia Centre *PSNR/SSIM*	Parameters
3DT-Net [58], 2021	Harvard, CAVE	**50.93/0.996**	**48.05/0.991**	-	-	3.46M
Fusformer [71], 2022	Harvard, CAVE	48.56/0.995	44.42/0.984	-	-	**0.10M**
Interactformer [72] 2022	-	-	-	29.74/0.9181	28.51/0.8897	4465 K

**Table 9:** Different Models of Image Super-Resolution for Different Datasets.

Method	Sub Task	Dataset	*PSNR/SSIM*	Parameters
TTSR [73], 2020	Reference-based SR ×4	CUFED5	27.09/0.804	9.10M
		Sun80	30.02/0.814	
		Urban100	25.87/0.784	
		Manga109	30.09/0.907	
CTCNet [75], 2022	Face Image SR ×8	CelebA	28.37/0.8115	-
		Helen	27.08/0.8077	
BN-CSNT [76], 2022	Thermal Image SR	PBVS-2022 ×2	21.08/0.7803	-
		PBVS-2022 ×4	33.64/0.9263	

**Table 10:** Image denoising.

Method	Train Dataset	Dataset	Noise Factor (NF)	*PSNR/SSIM*	Parameters
CVT [81], 2021	DIV2K	SET 12	15	34.548	-
			25	31.865	
			30	27.676	
		BSD68	15	33.790	
			25	30.828	
			30	26.688	
SwinIR [50], 2021	DIV2K	Kodak24	15	35.34	11.8M
			25	32.89	
			50	29.79	
		McMaster	15	35.61	
			25	33.20	
			50	30.22	
		Urban100	15	35.13	
			25	32.90	
			50	29.82	
		CBSD68	15	34.42	
			25	31.78	
			50	28.56	
IPT [59], 2021	ImageNet	CBSD68	30	32.32	46.0M
			50	29.88	
Uformer [86], 2022	SIDD and DND	SIDD	Real world Noise	39.89/0.960	50.88M
		DND	Real world Noise	40.04/0.956	
Restormer [43], 2022	DIV2K and SIDD	CBSD68	15	34.40	25.31M
			25	31.79	
			50	28.60	
		Urban100	15	35.13	
			25	32.96	
			50	30.02	
		SIDD	Real world Noise	40.02/0.960	
DnT [82], 2022	-	Set9	25	32.18	-
			50	29.29/	
			75	27.62/	
			100	26.31/	
		CBSD68	25	28.78/8.16	
			50	25.72/7.02	
TECDNet [85], 2022	-	SIDD	Real world Noise	39.77/0.970	20.87M
		DND	Real world Noise	39.92/0.955	
TC-Net [86], 2022	-	SIDD	Real world Noise	39.69/0.970	-
		DND	Real world Noise	39.88/0.954	
SUNet [87], 2022	DIV2K	CBSD68	10	35.94 0.958	99M
			30	30.28 0.870	
			50	27.85 0.799	
		Kodak24	10	36.79 0.953	
			30	31.82 0.899	
			50	29.54 0.810	
Pocoformer [88], 2022	Real-world polarized color image	Real-world polarized color image	Real world Noise	39.33/0.966	26.26M
TransCT [89], 2021	Mayo Clinic Low-Dose CT	Mayo	Real world Noise	0.923 ± 0.024	-
		Pig	Real world Noise	0.87 ± 0.029	
TED-Net [90], 2021	Mayo Clinic Low-Dose CT	Mayo	Real world Noise	/0.9144	18.88M
Eformer [91], 2021	Mayo Clinic Low-Dose CT	Mayo	Real world Noise	43.487/0.9861	-

**Table 11:** Image deblurring.

Method	Dataset Training	GoPro *PSNR/SSIM*	HIDE *PSNR/SSIM*	RealBlur-R *PSNR/SSIM*	Parameters
Uformer [86], 2022	GoPro	33.06/0.967	30.90/0.953	36.19/0.956	50.88M
Restormer [43], 2022	GoPro	32.92/0.961	31.22/0.942	36.19/0.957	25.31M
Multi-scale Cubic-Mixer [96], 2022	4KRD	33.79/0.962	-	39.66/0.969	40M
Stripformer [97], 2022	RealBlur	33.08/0.962	31.03/0.940	39.84/0.974	20M

**Table 12:** Image Dehazing.

Method	Dataset Training	SOTS-Indoor *PSNR/SSIM*	SOTS-Outdoor *PSNR/SSIM*	HSTS *PSNR/SSIM*	O-Hazy *PSNR/SSIM*	Parameters
DeHamer [99], 2022	RESIDE	36.63/0.9881	35.18/0.9860	-	-	-
TCAM [100], 2022	RESIDE	21.44/0.8851	-	23.83/0.9022	-	-
ISM [101], 2022	RESIDE	36.34/0.9836	30.85/0.9628	30.40/0.9696	-	-
Jiao et al. [102], 2022	O-hazy and I-hazy	-	-	-	15.89/0.56	-
Song et al. [103], 2022	RESIDE	38.46/0.994	34.29/0.983	-	-	4.634M
Zhao et al. [104], 2021	RESIDE	32.17/0.970	-	-	29.87/0.758	-

**Table 13:** JPEG compression artifact reduction.

Method	Type	Dataset	Quality Factor	*PSNR/SSIM*	Parameters
SwinIR [50], 2021	Single Image	Classic5	10	30.27/0.8249	11.8M
			20	32.52/0.8748	
			30	33.73/0.8961	
			40	34.52/0.9082	
		LIVE1	10	29.86/0.8287	
			20	32.25/0.8909	
			30	33.69/0.9174	
			40	34.67/0.9317	
PTNet [112], 2022	Pair Stereo images	Flickr1024	10	28.05/0.8403	0.91 M
			20	30.39/0.9017	
			30	31.83/0.9264	
		KITTI2012	10	31.43/0.8786	
			20	33.85/0.9231	
			30	35.18/0.9404	
		KITTI2015	10	31.42/0.8730	
			20	34.07/0.9245	
			30	35.57/0.9449	
		Middlebury	10	32.05/0.8676	
			20	34.51/0.9200	
			30	35.85/0.9400	

**Table 14:** Removing Adverse Weather Conditions.

Method	Dataset Training	Rain100L *PSNR/SSIM*	Rain100H *PSNR/SSIM*	SPAD *PSNR/SSIM*	Raindrop800 *PSNR/SSIM*	Snow100K *PSNR/SSIM*	Parameters
ETDNet [132], 2021	Rain100L and Rain100H	41.09/0.986	32.35/0.9299	-	-	-	32.97M
SDNet [133], 2021	Rain100L and Rain100H	37.92/0.9843	28.26/0.8957	-	-	-	2.14M
IPT [59], 2021	ImageNet	-	41.62/0.9880	-	-	-	46.0M
Uformer [86], 2022	SPAD	-	-	47.84/0.9925	-	-	50.88M
Restormer [43], 2022	Rain100L and Rain100H	38.99/0.978	31.46/0.904	-	-	-	25.31M
TAPE [134], 2022	Rain200H, Raindrop800 and Snow100K	33.17	-	-	27.69	26.33	1.07M
TransWeather [135], 2022	Raindrop800 and Snow100K	-	-	-	34.55/0.9502	33.78/0.9287	31 M
SiamTrans [136], 2022	NTURain	27.02/0.9024	-	-	-	26.05/0.8605	-

**Table 15:** Image Restoration and Enhancement.

Method	Type	Training Dataset		*PSNR/SSIM*	Parameters
RFormer [137], 2022	Medical Real Fundus restoration	120 pair Real Fundus		28.38/0.863	21.11M
UIE-IPT [139], 2022	Underwater Images	UFO-120		23.14/0.90	46.0M
FRGAN [140], 2021	Face Restoration	VGGFace2 and CASIA-WebFace		23.54/0.8199	-
DocEnTr [141], 2022	Enhance Handwritten Document Images	DIBCO		-/20.81	-
		H-DIBCO		-/22.29	
STUNet [142], 2022	Blind Face Restoration	EDFace-Celeb-1M (BFR128)		24.5500/0.6978	-
		EDFace-Celeb-150K (BFR512)		27.1833/0.7346	
DuDoTrans [143], 2021	Medical CT Reconstruction	NIH-AAPM		32.68/0.9047	-
		COVID-19		37.83/0.9727	
U2-Former [144], 2021	Image Reflection Removal	PLNet	Real20	23.67/0.835	-
			Nature	24.75/0.848	
			Solid	25.27/0.907	
			Wild	25.68/0.905	
			Postcard	22.43/0.889	
	Image Rain Removal	Rain100L		39.31/0.982	-
		Rain100H		30.87/0.899	
	Image Hazing Removal	RESIDE	Indoor	36.42/0.988	-
			Outdoor	31.10/0.976	
SMIR [145], 2021	MRI image reconstruction different sampling ratios	HCP	10%	-/0.72	11M
			20%	-/0.86	
			30%	-/0.87	
			40%	-/0.89	
			50%	-/0.91	
Wang et al. [146], 2021	Image Reconstruction	ImageNet 20k	Set5	-/32.61	-
			Set14	-/28.92	
			B100	-/27.78	
			Urban100	-/26.82	

## Data Availability

Not applicable.
